# Chronic kidney disease diagnosis using decision tree algorithms

**DOI:** 10.1186/s12882-021-02474-z

**Published:** 2021-08-09

**Authors:** Hamida Ilyas, Sajid Ali, Mahvish Ponum, Osman Hasan, Muhammad Tahir Mahmood, Mehwish Iftikhar, Mubasher Hussain Malik

**Affiliations:** 1 School of Electrical Engineering and Computer Science, National University of Sciences and Technology, H/12 Sector, Islamabad, Pakistan; 2 Department of Computer Science, Institute of Southern Punjab, Multan, Pakistan; 3 Department of Information Sciences, University of Education, Mulatan Campus, Lahore, Pakistan; 4 Department of Computer Science, University of Engineering and Technology, Taxila, Pakistan; 5 Department of Endocrinology and Metabolism, Services Hospital, Lahore, Pakistan

**Keywords:** CKD, GFR, Machine learning, Decision tree, J48, Random Forest

## Abstract

**Background:**

Chronic Kidney Disease (CKD), i.e., gradual decrease in the renal function spanning over a duration of several months to years without any major symptoms, is a life-threatening disease. It progresses in six stages according to the severity level. It is categorized into various stages based on the Glomerular Filtration Rate (GFR), which in turn utilizes several attributes, like age, sex, race and Serum Creatinine. Among multiple available models for estimating GFR value, Chronic Kidney Disease Epidemiology Collaboration (CKD-EPI), which is a linear model, has been found to be quite efficient because it allows detecting all CKD stages.

**Methods:**

Early detection and cure of CKD is extremely desirable as it can lead to the prevention of unwanted consequences. Machine learning methods are being extensively advocated for early detection of symptoms and diagnosis of several diseases recently. With the same motivation, the aim of this study is to predict the various stages of CKD using machine learning classification algorithms on the dataset obtained from the medical records of affected people. Specifically, we have used the Random Forest and J48 algorithms to obtain a sustainable and practicable model to detect various stages of CKD with comprehensive medical accuracy.

**Results:**

Comparative analysis of the results revealed that J48 predicted CKD in all stages better than random forest with an accuracy of 85.5%. The study also showed that J48 shows improved performance over Random Forest.

**Conclusions:**

The study concluded that it may be used to build an automated system for the detection of severity of CKD.

**Supplementary Information:**

The online version contains supplementary material available at 10.1186/s12882-021-02474-z.

## Background

The kidney is one of the most important body organs that filtrates all the wastes and water from human body to make urine. Chronic Kidney Disease (CKD), also commonly known as chronic renal disease or chronic kidney failure, is a life-threatening disease that is attributed to the failure of the kidney in performing its routine functionality. It leads to the continuous decrease of Glomerular Filtration Rate (GFR) for a period of 3 months or more and is a universal health problem. Some common symptoms of the disease include hypertension, irregular foamy urine, vomiting, shortness of breath, itching and cramps [1], whereas high blood pressure and diabetes are the main causes of this disorder.

CKD is often diagnosed in later stages when dialysis or kidney transplant are the only options left to save the patient’s life. Whereas an early diagnosis can lead to the prevention of kidney failure [2]. The best way to measure the kidney function or to predict the stages of kidney disease is to monitor the Glomerular Filtration Rate (GFR) on regular basis [3]. GFR is calculated using age, gender, race and blood creatinine value of a person. Based on the value of GFR, CKD may be categorized into six stages as shown in Table 1.

**Table 1:** CKD Stages According to GFR Measurement Values

Stage	GFR	Description
1	90–100 mL/min	Normal kidney function or structural abnormalities
2	60–89 mL/min	Mildly reduced kidney function
3A	45–59 mL/min	Moderately reduced kidney function
3B	30–44 mL/min	Moderately reduced kidney function
4	15–29 mL/min	Severely reduced kidney function
5	< 15 mL/min or dialysis	End stage kidney failure

Symptoms of CKD are not disease specific. The symptoms develop gradually, and some patients may not have any symptoms at all. Hence, it becomes very difficult to detect the disease at early stages.

Machine Learning (ML) has recently played a significant role for the diagnosis of diseases by just analyzing the records of existing patients and training a model to predict the behavior of new patients [3]. ML is a branch of Artificial Intelligence in which the computing machine learns automatically and thus the prediction gets better from training experiences. A category of ML is supervised learning which may be used for regression or classification of dataset. ML is being used very effectively in different domains, especially, in the biomedical field for the detection and classification of several diseases. Different ML algorithms may be used to predict diseases with each one having its own strength and weaknesses. Among these, decision-tree provides classified reports for kidney related diseases with more accuracy [3]. Thus, it seems quite suitable to be used to build a prediction system to diagnose kidney diseases at early stage.

CKD has been recognized as a leading public health issue. Millions of people die each year due to inadequate provision of healthcare, lack of health education [4] and high cost treatment of CKD. According to the global facts about kidney diseases, globally, 13.4% estimated population is affected by CKD [5]. Many studies have been conducted to predict the stages of CKD using different classification algorithms and acquired expected results of their proposed model. S. Ramya et al. [6] worked on Random Forest, Radial Basis Function and Back propagation Neural Network for the classification of CKD. The comparative study of three models revealed that Radial Basis Function provides 85.3% accuracy rate. Jing Xiao [7] established nine models and compared their performance to predict the CKD stages according to its severity. Predictive models include ridge regression, lasso regression, logistic regression, Elastic Net, XG Boost, neural network, k-nearest neighbor, random forest and support vector machine. Results of experiments obtained in their study, show that the Elastic net model produced the highest sensitivity, i.e., 0.85. Logistic regression provided the best results for sensitivity, specificity and Area Under the Curve (AUC) with 0.83, 0.82 and 0.873, respectively. El-Houssainy et al. [8] applied Probabilistic Neural Networks (PNN), Support Vector Machine (SVM) and Multilayer Perceptron (MLP) on the dataset to predict the severity of CKD. Their study resulted in a 96.7% classification accuracy, which is the highest derived by PNN with 12 s execution time, whereas, MLP had shown time efficiency and derived results with a minimum execution time of 3 s.

However, this study is significant, as not a single previous research is conducted to detect the stages of CKD using age, sex, race and Serum Creatinine attributes. In this study, we focus on using two machine learning algorithms i.e. J48 and Random Forest, to predict the stages of CKD. Our study reveals more accurate results than most of the existing studies, i.e., we achieved 85.5% accuracy using the J48 algorithm within 0.03 s and 78.25% accuracy using the random forest algorithm within 0.28 s.

## Methods

**Fig. 1:**
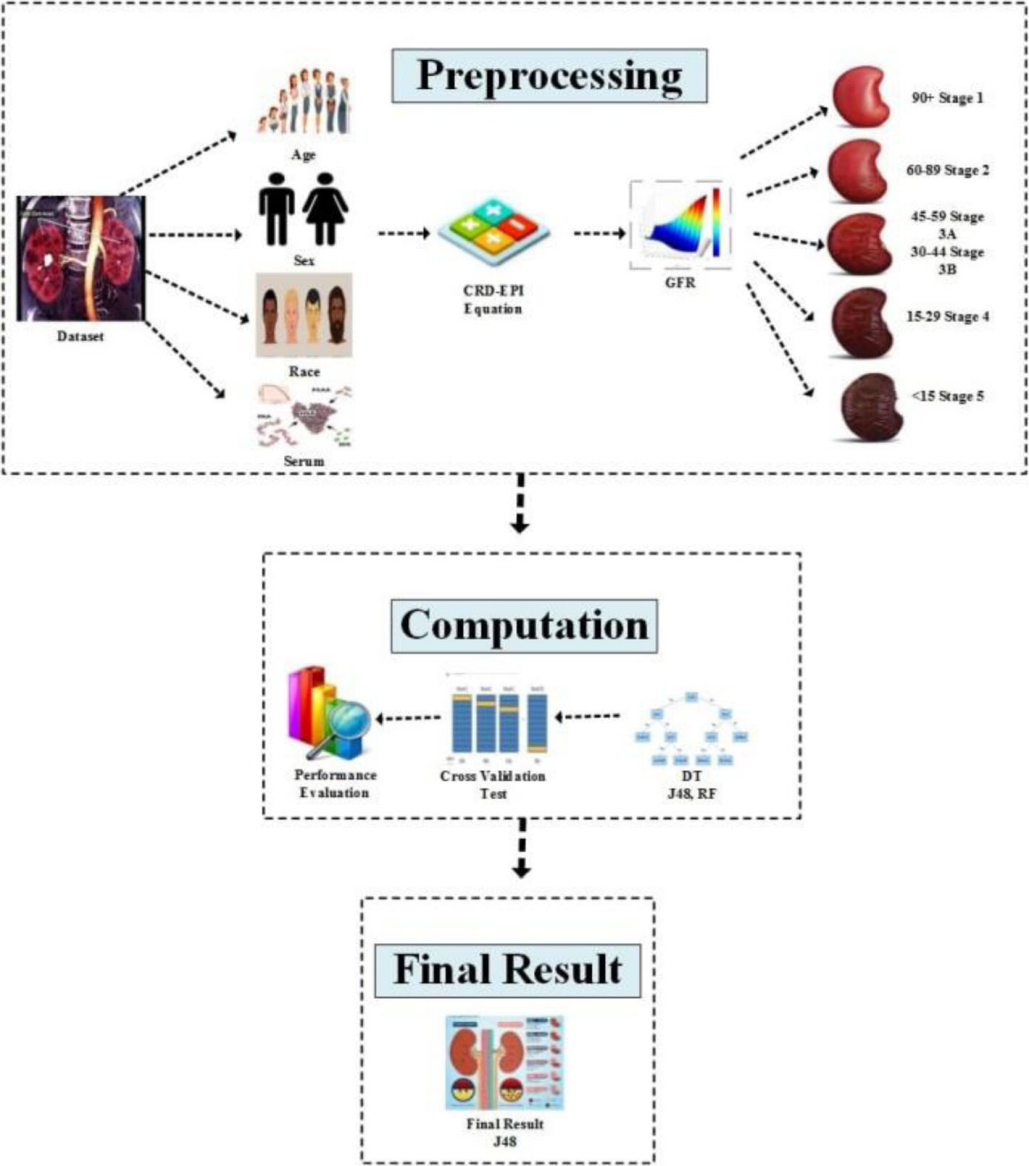
Block Diagram of Proposed Method Made in MS Visio 2013

This study reveals the results in three phases, i.e., preprocessing, computation and final results to predict the stages of chronic kidney disease. Block diagram of the proposed method is designed in MS Visio 2013 software by the authors, shown in Fig. 1. The methods were devised in accordance with relevant guidelines and regulations.

### Preprocessing

This phase starts from the acquisition of dataset of CKD patients. Four attributes, i.e., age, sex, race and serum creatinine, are selected from the dataset to be given as input in GFR calculation. Various mathematical equations are used for the estimation of GFR in the literature but we have chosen the Chronic Kidney Disease Epidemiology Collaboration (CKD-EPI) Equation [9], in this study to estimate GFR. As, this equation is reliable for the calculation of all stages of CKD as compared to Modification of Diet in Renal Disease (MDRD) Equation that relies only on serum creatinine, age gender and ethnicity and is known to be good only when GFR is > 60, which is the case for later stages of CKD.

#### Dataset

The dataset for the proposed system has been selected from the University of California Irvine (UCI) Machine Learning Repository, consisting of 400 instances and 25 attributes, which along with their description, their type and classes are given in Table 2. This dataset consists of only two classes, i.e., CKD affected and NOTCKD indicating people with no chronic kidney disease. The proposed system further subdivides the CKD class into different stages, i.e., Stage 1 represents normal kidney function, Stage 2 represents mildly reduced kidney function, Stage 3A represents moderately reduced kidney function, Stage 3B represents moderately reduced kidney function, Stage 4 represents severely reduced kidney function and Stage 5 represents end stage kidney failure of CKD using the calculated GFR values, as shown in Table 1.

**Table 2:** Variable Description Used in Analysis

Attribute Symbols and Description	Type	Class
age (Age)	Numerical	Predictor
bp (Blood Pressure)	Numerical	Predictor
sg (Specific Gravity)	Nominal	Predictor
al (Albumin)	Nominal	Predictor
su (Sugar)	Nominal	Predictor
rbc (Red Blood Cells)	Nominal	Predictor
pc (pus Cell)	Nominal	Predictor
pcc (Pus Cell Clumps)	Nominal	Predictor
rc (Race)	Nominal	Predictor
bgr (Blood Glucose Random)	Numerical	Predictor
bu (Blood Urea)	Numerical	Predictor
sc (Serum Creatinine)	Numerical	Predictor
sod (Sodium)	Numerical	Predictor
pot (Potassium)	Numerical	Predictor
hemo (Hemoglobin)	Numerical	Predictor
pcv (Packed Cell Volume)	Numerical	Predictor
sex (Sex)	Nominal	Predictor
rc (Red Blood Cell Count)	Numerical	Predictor
htn (Hypertension)	Nominal	Predictor
dm (Diabetes Mellitus)	Nominal	Predictor
cad (Coronary Artery Disease	Nominal	Predictor
appet (Appetite)	Nominal	Predictor
pe (Pedal Edama)	Nominal	Predictor
ane (Anemia)	Nominal	Predictor
class (Class)	Nominal	Target

In Table 2, the attribute symbols and description shows all attributes extracted from data, type column shows the datatype of attributes, whereas class in third column of Table 2 is actually categorization of attributes of dataset i.e. two categories (1) predictor and (2) target. Predictor attributes will be used to predict target. Using all predictor attributes class/stage of chronic kidney disease will be predicted.

#### Hardware requirements

The hardware used for this study is consisted of intel® core™ i5, CPU 2.40GHz, RAM 4 GB, 64-bit operating system (x-64 based processor).

#### Glomerular filtration rate (GFR)

GFR is defined as the amount of plasma that is filtered by glomeruli per unit of time and is calculated by estimating the rate of clearance of a substance from plasma. It is considered as one of the best attributes to measure the level of kidney function and to determine the severity of CKD [3]. The GFR value is calculated using filtration markers, which is a kidney excreted substance. The clearance of filtration marker is then used in a formula to determine GFR. Various mathematical equations are being used for the estimation of GFR but the most widely used ones include the following: [10].

- Chronic Kidney Disease Epidemiology Collaboration (CKD-EPI) Equation
- Modification of Diet in Renal Disease (MDRD) Equation

##### CKD-EPI equation

The equation for CKD-EPI is written as follow [9]:

1 $$\mathrm{GFR}=141\ast \min\ \left(\mathrm{SCr}/\mathrm{k},1\right){.^{\alpha}}_{\ast}\max\ {\left(\mathrm{SCr},1\right)}^{-1.209}0.{993^{\mathrm{age}}}_{\ast }1.018\ \left(\mathrm{if}\ \mathrm{female}\right)$$

SCr in eq. 1, represents the serum creatinine and k is constant, it stands for Kappa. There are different values of k for male and female, i.e. k = 0.7 for female and k = 0.9 is for male.

##### MDRD equation

The equation for MDRD is written as follow [9]:

2 $$\mathrm{GFR}=175\ast {\mathrm{SCr}}^{-1.154}\ast {\mathrm{age}}^{-0.203}\ast 0.742\ \left(\mathrm{if}\ \mathrm{female}\right)$$

Chronic Kidney Disease Epidemiology Collaboration (CKD-EPI) is considered to be more precise for the estimation of the glomerular filtration rate (eGFR) than the modification of diet in renal disease (MDRD) [10]. So, in the proposed work, we have chosen the CKD-EPI equation for the calculation of GFR. Four parameters, i.e., sex, race, Serum Creatinine, and age, are given as input to the equation (CKD-EPI) to calculate the GFR of the corresponding person.

### Computation

Computational engine has been implemented in our work using the WEKA data mining tool [11]. Classification algorithms are compared using the performance measures of execution time and classification accuracy. Testing and validation of the model has been done with the 15-fold cross validation technique. Then, finally the performance evaluation of the classification is done.

#### Classification of algorithms

##### Binary/ binomial classification

In this type of classification, the problem consists of two values for the class variable. From the given two classes, the algorithms predict one of these. i.e. disease exists or not, a match may be detected or not.

##### Multiclass/ multinomial classification

This type of classification is used for problems where there are more than two classes or labels, i.e., [0 to K-1]. From the given K-1 classes, the classifier predicts one of all these.

In this study, multiclass J48 and Random Forest classifiers are used to classify CKD into different stages. The description of both algorithms and the related algorithm’s working is explained in following subsections.

#### J48 algorithm

J48 (C4.5) is the most commonly used decision tree algorithm that is an extension of Quinlan’s earlier ID3 Algorithm - known to have a reasonable accuracy rate in bio-medical applications [12, 13]. It has the capability to handle both numerical and categorical data [14]. It is also named as statistical classifier [15]. It is easy to implement and deals with both noise and missing values [16]. Also, the performance of J48 is not good for a small training set [16].

The working of J48 algorithm, used in this study, is based on the following steps to produce output [17]:

1. Choose the dataset as an input to the rule for process. To split categorical attributes, J48 works just as the ID3 algorithm.
2. Calculate the Normalized information gain for each feature.
3. The feature with the maximum information gain is chosen as the best attribute. An attribute with the maximum information gain is selected as the root node to create a decision tree.
4. Repeat the above-mentioned step until some stop criterion, to compute the information gain for each attribute and add that attribute as children node.

#### Random Forest algorithm

Random Forest is an algorithm that is used for supervised classification. It creates a forest of large number of trees to calculate the accuracy efficiently [18]. The accuracy for this classifier is directly proportional to the number of trees. The results produced by Random Forest, even without hyper-parameter tuning, are more reliable because of its flexibility. It is simple and works very efficiently especially when the size of data set is large. It retains the accuracy rate by recognizing outliers and anomalies. However, it is not very straightforward to implement and is computationally expensive [19]**.**

**Fig. 2:**
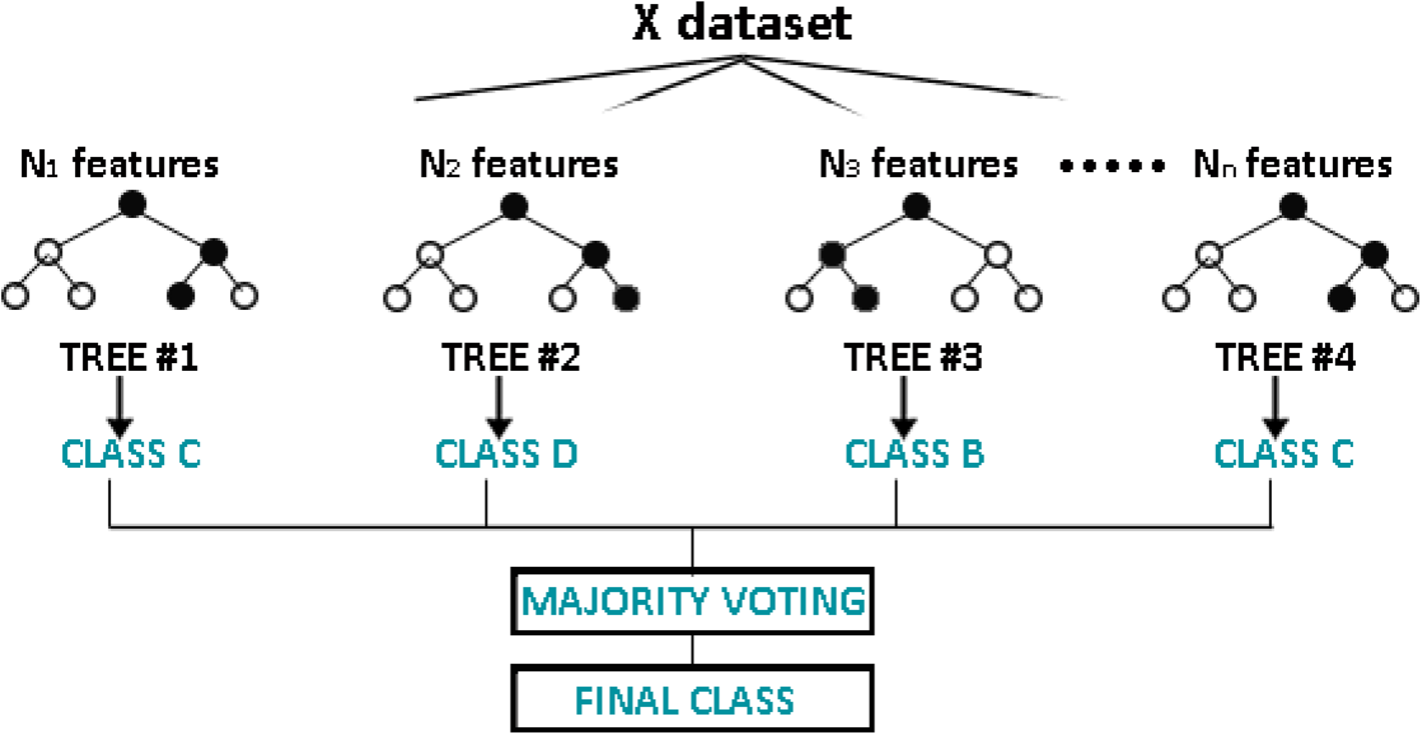
A Generalized Model of Random Forest

The working of Random Forest algorithm, used in this study, is based on the following steps to generate output:

1. Select samples randomly from the original dataset. Such kind of randomly selected samples are usually referred to as the bootstrapped data set.
2. Build a decision tree for the bootstrapped data set by considering a random subset of variables.
3. Repeat the above process 100 times (to the largest extent possible).
4. Predict the outcome for new data point by running the new data down all decision trees that are made.
5. The predicted class is judged based on the majority of votes.
6. Finally, evaluate the model by using the out of bag instances of the dataset to derive final class. A generalized model of the random forest algorithm is shown in Fig. 2.

#### Out of bag (OOB) instances

The instances which are not included in the bootstrapped data are termed as out of bag (OOB) instances. They, usually, form one third of the original dataset and are used to check the accurateness of the model by comparing the percentage of OOB samples that are correctly classified [20].

#### Out-of-bag error

Percentage of OOB instances that are not classified correctly are termed as Out-Of-Bag Error.

#### Cross validation

**Fig. 3:**
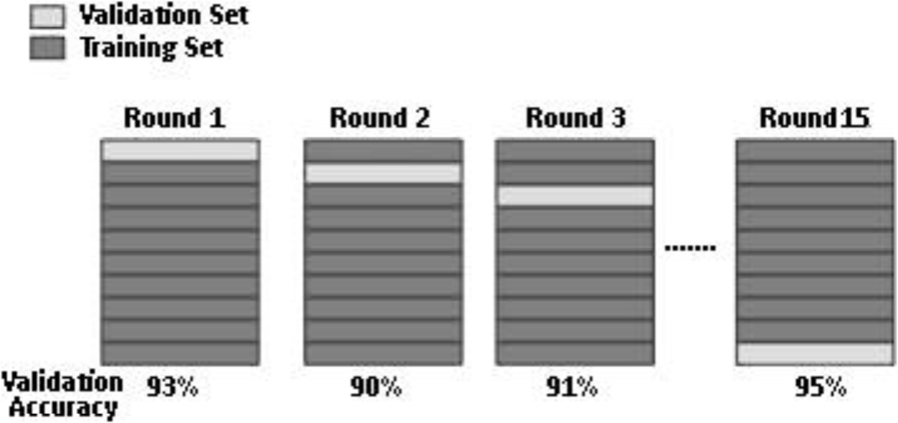
15-Fold Cross Validation

This method, used for model validation, divides the data set into a number of k-folds (one test other training). One-fold is used to test the model build on other parts. Model is repeated by building and testing for each fold. Finally, the average of all k-test errors is calculated. In this study, 15-fold cross validation is used to estimate the performance of model on the dataset. The general procedure of 15-fold cross validation is shown in Fig. 3.

Figure 3 shows that the complete dataset is shuffled randomly first and then the dataset is split into 15 groups. For each group, 1 group is taken as the test dataset and the remaining groups as a training dataset. Model is fitted on the training set and evaluated on the test set. Evaluation scores are retained as 93% in Round 1, 90% in Round 2 and till 95% in round 15.

### Performance evaluation of classification

Performance of classification is evaluated by calculating accuracy, sensitivity, specificity, F-Measure, and confusion matrix using the corresponding mathematical relationships, described below.

#### Accuracy

One of the most frequently used classification performance measures is accuracy. It is the ratio between the correctly classified samples to the total number of samples. The formula to calculate accuracy, used in this study is written as follows:

3 $$\mathrm{accuracy}=\frac{\mathrm{TP}+\mathrm{TN}}{\mathrm{TP}+\mathrm{TN}+\mathrm{FP}+\mathrm{FN}}$$

Where, TP represents true positive values, TN represents true negative values, FP represents false positive values and FN represents false negative values.

#### Sensitivity

It is also called True Positive Rate (TPR), hit rate or recall. It represents the ratio of correctly classified positive instances to the total number of positive instances. The formula to calculate sensitivity, used in this study, is written as follows.

4 $$\mathrm{Sensitivity}=\frac{\mathrm{TP}}{\mathrm{TP}+\mathrm{FN}}$$

#### Specificity

It is also called True Negative Rate (TNR) or inverse recall. It measures the percentage of correctly classified negative instances to the total number of negative instances. The formula to calculate specificity, used in this study, is written as follows.

5 $$\mathrm{Specificity}=\frac{\mathrm{TN}}{\mathrm{TN}+\mathrm{FP}}$$

#### F-measure

F-Measure is calculated by taking the weighted average of sensitivity and precision values. The formula to calculate F-Measure, used in this study, is written as follows [21].

6 $$\mathrm{F}-\mathrm{Measure}=\frac{2\ast \mathrm{sensitivity}\ast \mathrm{precision}}{\mathrm{sensitivity}+\mathrm{precision}}$$

F-Measure uses the field of information retrieval for the estimation of classification performance [17].

#### Precision

Precision is defined as what proportion of positive identifications was actually correct. The formula to calculate precision, used in this study, is written as follows.

7 $$\mathrm{Precision}=\frac{\mathrm{TP}}{\mathrm{TP}+\mathrm{FP}}$$

#### Confusion matrix

The confusion matrix is a tabular representation of predictions made by a model. It shows a number of incorrect and correct predictions. These are calculated by comparing the classification results n-test data. The representation of the matrix is in the form of x-by-x, where, x is the number of classes in the dataset. Confusion matrix is a very strong tool to calculate the accuracy of a classifier [10].

In Table 3, TP_A_ represents the true positive values, which means that they predicted values correctly predicted as actual positive values in class A. TP_B_ represents that the predicted values correctly predicted as actual positive values in class B. TP_C_ represents the true positive values, which means that predicted values correctly predicted as actual positive values in class C. E_AB_ are the samples of class A which are misclassified as B. E_AC_ are the samples of class A which are misclassified as *C. E*_*BA*_ are the samples of class B which are misclassified as A. E_BC_ are the samples of class B which are misclassified as C. E_CA_ are the samples of class C which are misclassified as A. E_CB_ are the samples of class C which are misclassified as B.

**Table 3:** Confusion Matrix for Multi-Class Classification

		True Class		
Predicted Class		A	B	C
	A	TP_A_	E_BA_	E_CA_
	B	E_AB_	TP_B_	E_CB_
	C	E_AC_	E_BC_	TP_C_

In Tables 4 and 5, a represents CKD Stage 2 (mildly reduced kidney function), b represents CKD Stage 1 (normal kidney function or structural abnormalities), C represents CKD stage 3B (moderately reduced kidney function),D represents CKD stage 4 (severely reduced kidney function), E represents CKD stage 3A (moderately reduced kidney function), F represents CKD Stage 5 (end stage kidney failure). FN_A_ is False Negative in class A. FN_A_ is calculated by using the formula FN_A_ = E_AB_ + E_AC_. FP_A_ is False Positive in class A and calculated by using the formula FP_A_ = E_BA_ + E_CA_.

**Table 4:** Confusion Matrix for J48

	a	b	c	d	e	f	
**a**	**9**	**2**	**0**	**0**	**4**	**0**	**FP**_**a**_ **= 15**
**b**	**3**	**12**	**0**	**0**	**0**	**0**	**FP**_**b**_ **= 15**
**c**	**0**	**0**	**28**	**9**	**2**	**2**	**FP**_**C**_ **= 41**
**d**	**0**	**0**	**7**	**50**	**0**	**1**	**FP**_**d**_ **= 58**
**e**	**3**	**0**	**6**	**0**	**21**	**0**	**FP**_**e**_ **= 30**
**f**	**1**	**1**	**3**	**6**	**2**	**72**	**FP**_**f**_ **= 85**
	**FN**_**A**_ **= 16**	**FN**_**b**_ **= 15**	**FN**_**c**_ **= 44**	**FN**_**d**_ **= 59**	**FN**_**e**_ **= 28**	**FN**_**f**_ **= 75**

**Table 5:** Confusion Matrix for Random Forest

	a	b	c	d	e	f	
**a**	3	2	0	0	5	5	**FPa = 15**
**b**	3	9	0	0	0	3	**FP**_**b**_ **= 15**
**c**	0	0	23	5	2	11	**FP**_**C**_ **= 41**
**d**	0	0	4	42	0	12	**FP**_**d**_ **= 58**
**e**	0	0	12	0	11	7	**FP**_**e**_ **= 30**
**f**	1	1	2	6	1	75	**FP**_**f**_ **= 86**
	**FN**_**A**_ **= 7**	**FN**_**b**_ **= 12**	**FN**_**c**_ **= 41**	**FN**_**d**_ **= 53**	**FN**_**e**_ **= 19**	**FN**_**f**_ **= 113**	

## Results

Results were derived for CKD Stage 1 (normal kidney function or structural abnormalities), Stage 2 (mildly reduced kidney function), Stage 3A (moderately reduced kidney function), Stage 3B (moderately reduced kidney function), Stage 4 (severely reduced kidney function) and Stage 5 (end stage kidney failure).

Table 6 provides the summary of classification results of the CKD patients with Stage 1 using j48 and random forest algorithm. An accuracy of 96% using j48 and random forest algorithm was achieved. The j48 algorithm exhibited a sensitivity of 56% whereas the random forest algorithm exhibited a sensitivity of 43%. Similarly, 98% specificity was achieved using j48 algorithm and 96% with random forest algorithm. Precision, recall, F-Measure and ROC area was obtained as 0.56, 0.52, 0.55 and 0.86, respectively, using j48 algorithm and 0.429, 0.176, 0.250, 0.947, respectively, using the random forest algorithm. J48 revealed better results than random forest algorithm to predict the kidney performing normal function.

**Table 6:** Summary of algorithms classification outputs for classifying the Chronic Kidney Disease patients with stage 1

	J48	Random Forest
Total instances	400	400
True Positive (TP)	9	3
True Negative (TN)	376	379
False Positive (FP)	8	14
False Negative (FN)	7	4
Accuracy	96%	96%
Sensitivity	56%	43%
Specificity	98%	96%
Precision	0.56	0.429
Recall	0.52	0.176
F-Measure	0.55	0.250
ROC Area	0.86	0.947

The summary of classification results of the CKD patients with Stage 2 using j48 and random forest algorithm is given in Table 7. An accuracy of 96 and 93% was achieved using j48 and random forest algorithms, respectively. Sensitivity of 72 and 58% was gained using j48 algorithm and random forest algorithm, respectively. Similarly, specificity 98 and 95% was achieved using j48 algorithm and the random forest algorithm, respectively. Precision, recall, F-Measure and ROC area was obtained as 0.72, 0.70, 0.71 and 0.93, respectively, using j48 algorithm and 0.579, 0.367, 0.449, 0.958, respectively, using the random forest algorithm. Thus, in the prediction of CKD Stage 2 (mildly reduced kidney function), J48 revealed better results than random forest algorithm.

**Table 7:** Summary of algorithms classification outputs for classifying the Chronic Kidney Disease patients with stage 2

	J48	Random Forest
Total Instances	400	400
True Positive (TP)	21	11
True Negative (TN)	362	362
False Positive (FP)	9	19
False Negative (FN)	8	8
Accuracy	96%	93%
Sensitivity	72%	58%
Specificity	98%	95%
Precision	0.72	0.579
Recall	0.70	0.367
F-Measure	0.71	0.449
ROC Area	0.93	0.958

Table 8 summarizes the results of classification of the CKD patients with Stage 3A using j48 and random forest algorithms. An accuracy of 98% using j48 and random forest algorithm was achieved. The j48 algorithm exhibited a sensitivity of 80% whereas the random forest algorithm exhibited a sensitivity of 75%. Similarly, 99% specificity was achieved using j48 algorithm and 98% with random forest algorithm. Precision, recall, F-Measure and ROC area was obtained as 0.80, 0.75, 0.77 and 0.92, respectively, using j48 algorithm and 0.75, 0.56, 0.64, 0.99, respectively, using the random forest algorithm. The Stage 3A (Moderately reduced kidney function) of CKD was predicted efficiently with more accuracy, sensitivity and specificity using j48 algorithm.

**Table 8:** Summary of algorithms classification outputs for classifying the Chronic Kidney Disease patients with stage 3A

	J48	Random Forest
Total instances	400	400
True Positive (TP)	12	9
True Negative (TN)	381	381
False Positive (FP)	4	7
False Negative (FN)	3	3
Accuracy	98%	98%
Sensitivity	80%	75%
Specificity	99%	98%
Precision	0.80	0.75
Recall	0.75	0.56
F-Measure	0.77	0.64
ROC Area	0.92	0.99

Table 9 provides the summary of classification results of the CKD patients with Stage 3B using j48 and random forest algorithms. An accuracy of 94 and 93% was achieved using j48 and random forest algorithms, respectively. Sensitivity of 77 and 79% was gained using j48 algorithm and random forest algorithm, respectively. Similarly, specificity 98 and 95% was achieved using the j48 algorithm and random forest algorithm, respectively. Precision, recall, F-Measure and ROC area was obtained as 0.78, 0.86, 0.81 and 0.96, respectively, using j48 algorithm and 0.792, 0.724, 0.757, 0.973, respectively, using the random forest algorithm. Thus, the performance of the J48 is more effective than the random forest algorithm to predict Stage 3B (Moderately reduced kidney function) of CKD.

**Table 9:** Summary of algorithms classification outputs for classifying the Chronic Kidney Disease patients with stage 3B.

	J48	Random Forest
Total instances	400	400
True Positive (TP)	50	42
True Negative (TN)	327	331
False Positive (FP)	8	16
False Negative (FN)	15	11
Accuracy	94%	93%
Sensitivity	77%	79%
Specificity	98%	95%
Precision	0.78	0.792
Recall	0.86	0.724
F-Measure	0.81	0.757
ROC Area	0.96	0.973

Table 10 provides the summary of classification results of the CKD patients with Stage 4 using j48 and random forest algorithms. An accuracy of 95 and 87% was achieved using the j48 and the random forest algorithm, respectively. Sensitivity of 96 and 66% was gained using the j48 algorithm and the random forest algorithm, respectively. Similarly, specificity of 95% was achieved using both the j48 and random forest algorithms. Precision, recall, F-Measure and ROC area was obtained as 0.96, 0.82, 0.88 and 0.95, respectively, using the j48 algorithm and 0.664, 0.852, 0.746, 0.938, respectively, using the random forest algorithm. Here also, J48 algorithm predicted the Stage 4 (Severely reduced kidney function) of CKD more accurately than the random forest algorithm.

**Table 10:** Summary of algorithms classification outputs for classifying the Chronic Kidney Disease patients with stage 4

	J48	Random Forest
Total instances	400	400
True Positive (TP)	72	75
True Negative (TN)	309	274
False Positive (FP)	16	13
False Negative (FN)	3	38
Accuracy	95%	87%
Sensitivity	96%	66%
Specificity	95%	95%
Precision	0.96	0.664
Recall	0.82	0.852
F-Measure	0.88	0.746
ROC Area	0.95	0.938

Table 11 summarizes the results of classification of the CKD patients with Stage 5 using the j48 and random forest algorithms. An accuracy of 93 and 91% was achieved using the j48 and the random forest algorithms, respectively. Sensitivity of 64 and 56% was gained using the j48 algorithm and the random forest algorithms, respectively. Similarly, specificity 96 and 95% was achieved using the j48 algorithm and the random forest algorithm, respectively. Precision, recall, F-Measure and ROC area was obtained as 0.64, 0.68, 0.66 and 0.91, respectively, using the j48 algorithm and 0.561, 0.561, 0.561, 0.914, respectively, using the random forest algorithm. The Stage 5 (End stage kidney failure) of CKD is also predicted more efficiently using J48 than random forest algorithm.

**Table 11:** Summary of algorithms classification outputs for classifying the Chronic Kidney Disease patients with stage 5

	J48	Random Forest
Total instances	400	400
True Positive (TP)	28	23
True Negative (TN)	343	341
False Positive (FP)	13	18
False Negative (FN)	16	18
Accuracy	93%	91%
Sensitivity	64%	56%
Specificity	96%	95%
Precision	0.64	0.561
Recall	0.68	0.561
F-Measure	0.66	0.561
ROC Area	0.91	0.914

At the end, the overall performance of both algorithms was compared. J48 provided 85.5% overall accuracy within 0.03 s, whereas, random forest achieved 78.25% accuracy within 0.28 s, as shown in Table 12.

**Table 12:** Overall Accuracy and Execution Time of Algorithms

	J48	Random Forest
Overall accuracy	85.5	78.25
Total execution time (seconds)	0.03	0.28

**Fig. 4:**
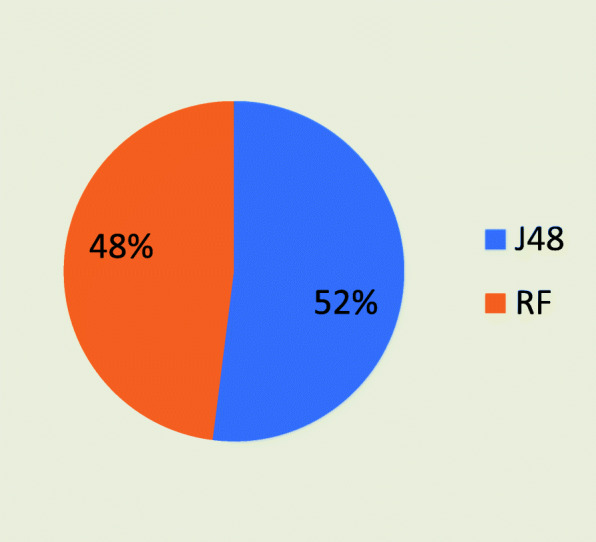
Comparison on the base of overall accuracy

Figure 4 shows the comparison of both algorithms with respect to the accuracy and execution time. Figure 4 shows that the J48 algorithm provided better results to diagnose the stages of CKD, as compare to random forest by providing an overall accuracy of 52%. Hence, based on the performance evaluation, J48 diagnosed all stages of CKD more accurately within less time than random forest.

## Discussion

Chronic Kidney Disease (CKD) refers to chronic disease associated with kidney failure. Traditionally, the kidney functioning is judged via blood and urine tests. However, it is important to develop a CKD screening system to identify the early stages of CKD and its symptoms. So that the preventive measures can be taken to alleviate the disease at an early stage and to avoid its complications.

**Fig. 5:**
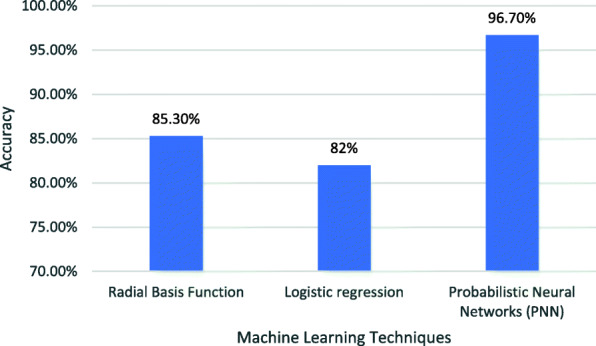
Comparison of studies on the base of overall accuracy

Machine Learning (ML) algorithms can be used to make reasonable accurate decisions when relevant data is given. Various studies have been conducted to detect CKD by using different parameters including age, sex, estimated GFR, serum calcium etc. S. Ramya et al. used radial basis function in their study to predict CKD using R language [6]. They used medical reports of patients collected from different laboratories as an input dataset. Their study obtained 85.3% accuracy to detect CKD. In 2019, Jing Xiao conducted a study to detect various stages of CKD [7]. This study used the logistic regression machine learning technique to train the model and used online tool for prediction. The authors further used medical records of patients in Shanghai Huadong Hospital as input dataset. This study obtained 85% accuracy to detect CKD. Later, in 2019, El-Houssainy et al. [8] used the UCI repository data to train the model using the DTREG predictive modeling system. They revealed the results using a probabilistic neural network and obtained 96.7% accuracy within 12 s. More details about the above-mentioned studies is shown in Table 13 and graph of accuracies is shown in Fig. 5.

**Table 13:** Detailed Information of Various Studies

Machine Learning Technique	Year	Author	Resources of Data Set	Disease	Tool	Accuracy	Execution Time in seconds
Radial Basis Function	2016	S. Ramya et al.	Medical reports of patients collected from different laboratories	Chronic Kidney Disease	R	85.3%.	N/A
Logistic regression	2019	Jing Xiao	Medical record of patients in Shanghai Huadong Hospital	Chronic Kidney Disease	online tool	82%	N/A
Probabilistic Neural Networks (PNN)	2019	El-Houssainy A. Radya, Ayman S. Anwar	University of California Irvine (UCI) Machine Learning Repository	Chronic Kidney Disease	DTREG Predictive Modeling System	96.7%	12

This study achieved 85.5% accuracy within 0.03 s. Although, the performance efficiency is less than the PNN, shown in Fig. 5, but time efficiency is better than PNN. When large amount of data is provided, the performance of ML algorithms usually improves in terms of accuracy. In this study, although we used a relatively small dataset, the sample size satisfied the analysis and concluded that the J48 algorithm performed better than the random forest algorithm. If large dataset is used then it is expected that J48 will perform better than PNN too. Our research work shows that stages of CKD can be predicted and classified with reasonable accuracy using ML classification techniques within less time as compared to the studies shown in Table 13. Results of Table 6, 7, 8, 9, 10, 11, 12 show that J48 provides better accuracy rate, precision and higher F-Measure as compared to Random Forest for classifying CKD into stages according to severity.

## Conclusion

In this study, we established and compared two algorithms including J48 and random forest to predict the various stages of CKD. It is observed that the ratio of correctly classified instances by J48 is 85.5%, whereas, it is 78.25% for Random Forest. On the other hand, the time taken by J48 is 0.03 s and for Random forest it is 0.28 s. Hence, it can be said that J48 is accurate and efficient in terms of execution time because its comparison with Random Forest shows that it provides results with better accuracy and less time.

J48 performs better than Random forest because it deals with both categorical and continuous values, whereas Random forest gets biased in favor of the attributes with categorical values. Random forest builds multiple decision trees, merges them together to get a stable prediction model. But this approach makes the algorithm slow and ineffective for real time-prediction. J48 is easy to implement but Random forest is hard to implement because of large number of trees. So, based on our results, we recommend using j48 to help physicians in generating an automated decision support system for diagnosing CKD.

## Supplementary Information

**Additional file 1.**

## Data Availability

Data is available as a supplementary file. The code and Weka file will be provided to each reader on demand. Reader can request via email from the corresponding author.

## Abbreviations

CKD: Chronic kidney disease
GFR: Glomerular filtration rate
CKD-EPI: Chronic kidney disease epidemiology collaboration
AUC: Area under the curve
PNN: Probabilistic neural networks
SVM: Support vector machine
MLP: Multilayer perceptron
MDRD: Modification of diet in renal disease
UCI: University of California irvine
SCr: Serum creatinine
OOB: Out of bag
TPR: True positive rate
